# Delusional Infestation Secondary to the Dopamine Agonist Ropirinole in a Patient With Parkinson's Disease: A Case Report With an Outline of the Biology of Psychosis

**DOI:** 10.7759/cureus.12880

**Published:** 2021-01-23

**Authors:** Hassan Kesserwani

**Affiliations:** 1 Neurology, Flowers Medical Group, Dothan, USA

**Keywords:** dopamine agonist, psycho-behavioral

## Abstract

Delusional infestation (DI) is a thought disorder, a delusion that one is infested with pathogens. Remarkably, these patients do not typically exhibit symptoms of anxiety or depression. The role of the dopamine D2 receptor is central to the idea of psychosis. In this article, we present a case of ropirinole-induced delusional skin infestation in a patient with Parkinson's disease, that was reversible with drug discontinuation. We seize upon this opportunity to discuss the pathology of the dopamine receptors, the glutamate N-methyl D-aspartate (NMDA) receptors and the serotonin 5-hydroxytryptamine (5HT-2A) receptors in the generation of psychosis. We outline the fundamental pharmacodynamical differences between the typical and atypical anti-psychotics that will help us understand how these agents work favorably and adversely. We also briefly review the neuroradialogy of psychosis and adumbrate on the mismatch between the meso-limbic system (motivational) and the salience network (valence) as the driver of the psychotic phenomenon.

## Introduction

Delusional infestation (DI), also referred to as delusional parasitosis, is a psychotic disorder characterized by a delusional belief of invasion of one's skin by a foreign agent, typically an insect (microscopic) and infrequently, inanimate objects. It is a haptic (tactile) disorder that is not generated by a pathology of the skin but by a thought disorder. DI can be solitary, a pure disorder (sui generi) or secondary to underlying psychopathology or iatrogenic. The intensity of this delusional disorder can lead to excoriation of the skin due to excessive scratching. The typical patient is an elderly woman with a vascular dementia or other neurodegenerative dementia, usually with multiple co-morbid conditions. When the young are afflicted, one has to consider drug use with dopamine-modulating agents, such as cocaine, amphetamines, or marijuana derivatives [1].

In order to understand the pathophysiology of the dopaminergic system, we need to distinguish between the first and second-generation neuroleptics (dopamine antagonists). The first generation (typicals) anti-psychotics are mostly effective against the positive symptoms, delusions and hallucinations, of schizophrenia. The second-generation (atypicals) anti-psychotics may do better with the negative symptoms, apathy and blunted effect. They both block the dopamine D2 receptors of the striatal system, leading to Parkinsonism and tardive dyskinesia, probably less so with the atypicals. The atypicals also block serotonin, 5-hydroxytryptamine (5HT-2A) receptors and have a higher affinity for the dopamine D4 receptor in the meso-limbic system, which is thought to improve negative symptoms [2]. 

The pathophysiology is believed to be dopamine-driven. The DI-inducing agents listed above are pre-synaptic dopamine transporter (DAT) inhibitors that increase synaptic dopamine. Furthermore, withdrawing the offending agent in primary DI usually resolves the condition. In DI secondary to psychopathology (secondary DI), dopamine D2 receptor blockers such as haloperidol are effective. The second-generation neuroleptics, such as quetiapine, which favor dopamine D3/D4 receptor blockade are less effective. However, randomized controlled trials are lacking [3,4]. 

Not surprisingly, lesions in the dopamine-rich putamen have been described in a patient with primary DI [5].

DI has been described in Parkinson's disease patients treated with dopamine agonists, such as ropirinole, with a resolution of symptoms after medication withdrawal. DI has also been associated with amantadine, which has both dopaminergic and anti-cholinergic activity [6].

## Case presentation

An 83-year-old woman with Parkinson's disease treated with carbidopa-levodopa (25/100) milligrams (mg), one tablet four times daily, was given a rapid titration of ropirinole, target dose 3 mg nightly for the restless legs syndrome. Over the course of seven months, she slowly developed the unshakeable belief that there were ants and splinters of wood invading the skin of the arms and legs. This belief was accompanied by vigorous and continual scratching of the skin of the arms and legs, to the point of excoriation and scab formation. A careful examination by a dermatologist did not reveal any primary skin disease. A short course of prednisone did not afford her any relief. Neither did the application of over-the-counter anti-itch ointments. Interestingly, she did not display any symptoms of anxiety or depression. The patient calmly acknowledged the presence of ants crawling on her skin both visually and by tactile sensation. There was no report of an impulse control disorder such as gambling, excessive spending, binge-eating, alcohol or drug abuse, or hypersexuality.

Past medical history was significant for atrial fibrillation treated with apixaban 2.5 mg twice daily.

On examination, she was calm and conversed fluently. Speech was mildly hypophonic. Her attention as measured by digit span, verbal fluency, visuo-motor skills with pantomime and delayed recall revealed no significant cognitive abnormalities. Occasionally she would scratch her forearm gently and acknowledged the presence of ants on her skin. Her gait revealed unsteadiness with mild retropulsion and an absent Romberg sign. Motor examination revealed an " on-state " with mild axial dyskinesias. Sequence motion of the fingers was symmetrically diminished without cog-wheeling (on-state). The rest of the neurological examination including cranial nerves, power and cerebellar function was normal. There was no evidence of a myelopathy. Deep tendon reflexes were normal except for absent ankle jerks and vibration sense was diminished in the toes, not unusual for an 83-year-old.

**Figure 1:**
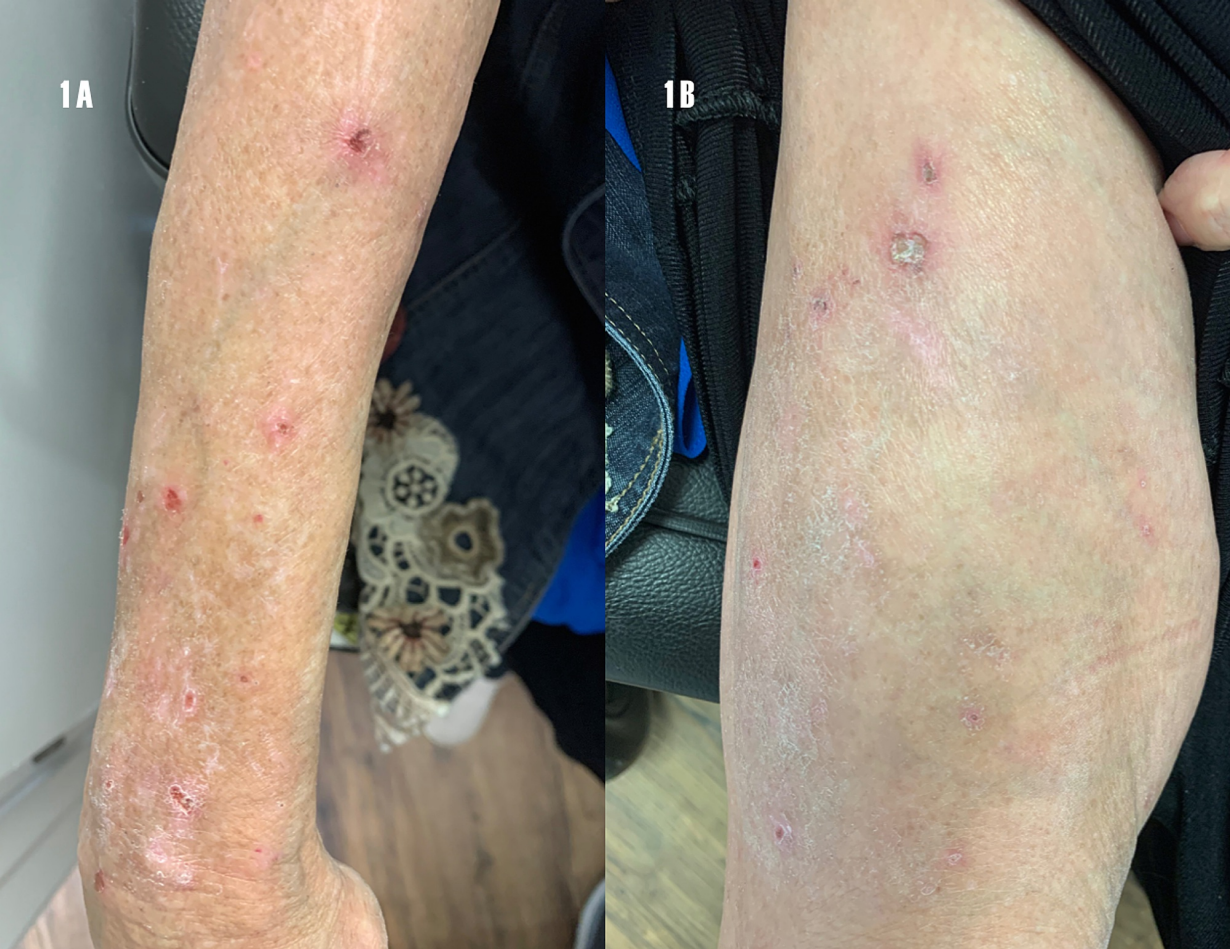
Excoriations and abrasions. (1A) Right extensor arm; (1B) right thigh.

The most pertinent feature on examination is the skin examination of the limbs, revealing abrasions, scabs and excoriations (Figure 1).

The patient was advised to slowly wean off ropirinole. Instead, her husband stopped ropirinole immediately, and the skin scratching started abating by 24 hrs and there was a complete resolution by 72 hours. 

## Discussion

There are at least five dopamine receptor sub-types. Their location, pharmacodynamics and dysfunction are listed below, the D5 receptor is omitted as it is not relevant to our discussion (Table 1) [7]. 

**Table 1:** Brief overview of dopamine pharmacology. Dopamine (D), dopamine-regulated neuronal phosphoprotein (DARP), N-methyl D-aspartate (NMDA), neuronal receptor (NR), 5-hydroxy-tryptamine (5HT), nucleus accumbens (NA), substantia nigra pars compacta (SNpc), substantia nigra pars reticularis (SNpr).

Receptor sub-type	D1	D2	D3	D4
Anatomy	Striatum (caudate, putamen), NA, SNpr, frontal and temporal cortex	SNpc, ventral tegmental nucleus, pituitary, olfactory bulb	Striatum, NA, hypothalamus	Frontal cortex, hypothalamus, amygdala, NA
Agonist action	Locomotion, attention	Locomotion, attention	Cognition, attention, impulse control	Cognition, attention, impulse control
Dysfunction	Dyskinesia	Extra-pyramidal syndrome, psychosis		
Mechanism	DARP 32 polymorphism/NMDA/NR1/NR2 subunit trafficking	Heteromeric complexing of adenosine receptors - downregulation of D2 receptor / prolactin elevation	Pre-synaptic: modulates dopamine release	5HT-2A antagonism high D4 receptor blockade accounts for clozapine activity

**Figure 2:**
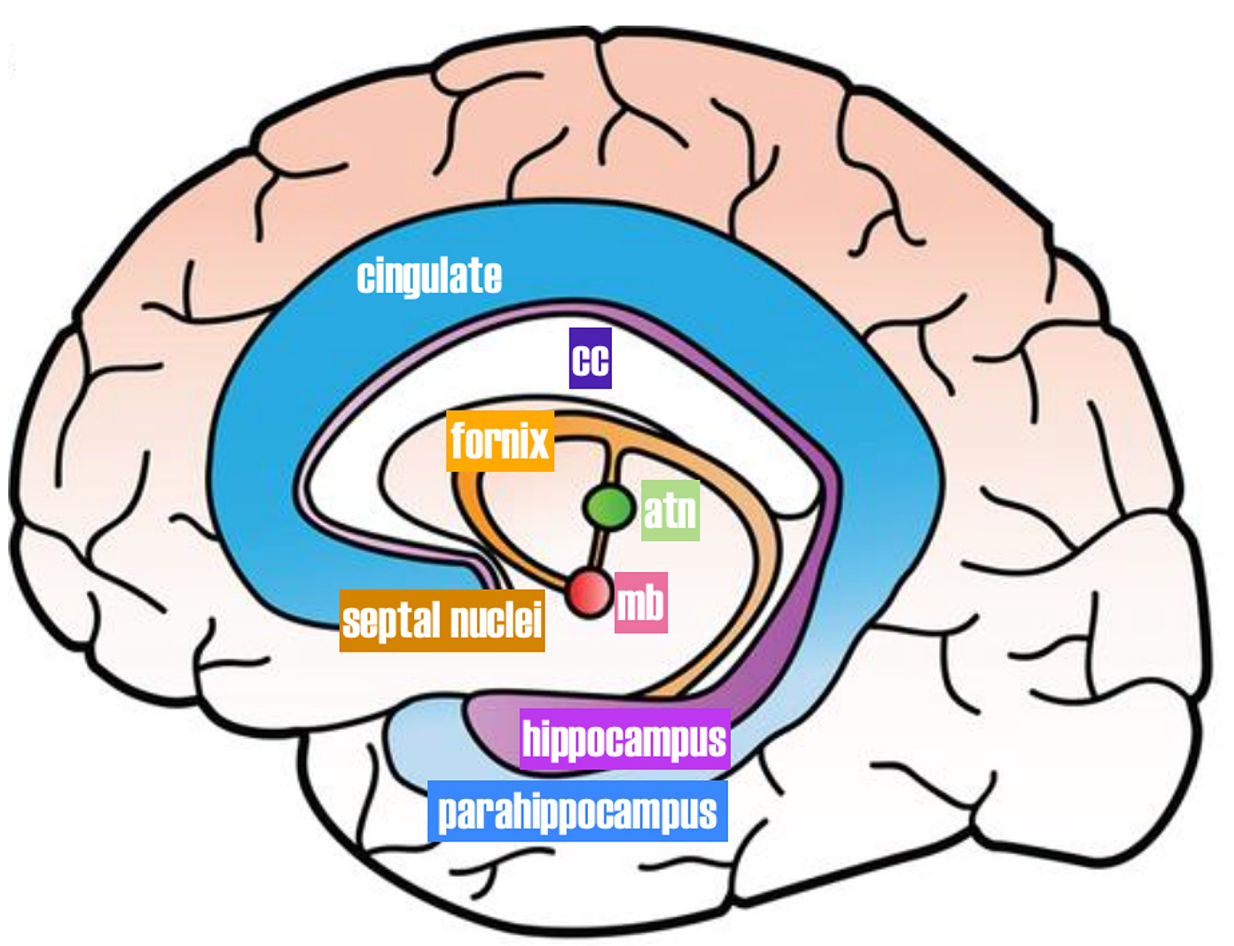
Papez circuit (limbic system). The septal nuclei are part of the limbic forebrain. Corpus callosum (cc), mammillary body (mb), anterior thalamic nucleus (atn).

We emphasize the localization of the D4 and 5HT-2A receptors in the limbic system. The limbic system processes the emotional content or value of a signal or sensation. The limbic system involves the classic C-shaped circuit, the Papez circuit linking the hippocampus, fornix, anterior thalamus, hypothalamus, cingulate gyrus and basal forebrain. Here the emotional content and motivational measure (septal nuclei; nucleus accumbens) are matched to its pre-existing memory (mammillary bodies, fornix, hippocampus) and value or valence (anterior thalamic nucleus, cingulate cortex and insula) of the stimulus (Figure 2) [8].

The pharmacodynamics of the D2 receptor will help us understand the "dopamine hypothesis" of psychosis. We can think of the D2 receptor as the "psychosis receptor". The atypical anti-psychotics, such as clozapine and quetiapine, dissociate rapidly from the D2 receptor, in a matter of seconds, compared to the typical anti-psychotics, such as haloperidol whose binding kinetics last for at least 30 minutes. The short-half life of the kinetic binding of the atypicals explains their lower rates of extra-pyramidal side-effects. This is known as the fast-off-D2 property. There needs to be at least 65%-85 % binding of the D2 receptors for therapeutic efficacy. At this threshold, side effects such as Parkinsonism and hyper-prolactinemia emerge. Patients with schizophrenia are super-sensitive to dopamine-enhancing drugs, such as amphetamines, due to the high proportion of high-affinity D2 receptors. It turns out that the proportion and not the number of high-affinity D2 receptors underlies the "wind of fire of the psychosis" [9,10].

The balance between the efficacy of anti-Parkinsonian medications and side-effects is delicate. Up to 40% of patients with Parkinson's disease develop psychosis, delusions and far more frequently hallucinations; visual, auditory, tactile and olfactory, in descending order of frequency. Most anti-Parkinsonian medications can cause hallucinations including levodopa, dopamine agonists and amantadine. Advancing age, dementia and poly-pharmacy increase the risk. The table below shows that all the commonly used dopamine agents avidly bind to the dopamine D2-receptor (Table 2).

**Table 2:** Dopamine receptor binding of the commonly used dopamine agonists, highlighting avid binding to the dopamine D2-receptor. Dopamine (D).

Dopamine agonist	D1	D2	D3	D4
Ropirinole	0	+++	+++	0
Pramipexole	0	+++	+++	++
Bromocriptine	0	++	+	+
Apomorphine	0	++	++	+++

It is well known that cholinergic deficits of the basal forebrain, basalis nucleus of Meynert, and the use of anti-cholinergics can lead to psychosis. In fact, the use of anti-choIinergics is contra-indicated after the age of 70. Disruption of sleep hygiene and its fragmentation can lead to suppression of nocturnal rapid-eye-movement (REM) sleep. This can lead to daytime intrusion of REM sleep during the awake state and vivid visual hallucinations. This explains the rationale for the use of cholinesterase inhibitors (increasing synaptic acetylcholine) and benzodiazepines (REM suppressants) in psychosis [11]. 

The benefits of the selective 5-hydroxytryptamine (5HT-2A) inverse agonists, pimavanserin, has been well established for the treatment of the psychosis of Parkinson's disease. This drug is modeled on the action of the hallucinogen lyseric acid dethylamide (LSD), a 5HT-2A agonist [12].

Nineteen subjects with predisposition to psychosis were studied with voxel-based morphometry, an automated MRI technique for assessing structural changes in the brain. They displayed reduced gray matter volume in the limbic cortex (bilateral hippocampal/para-hippocampal gyri and bilateral anterior cingulate cortex), components of the circuit of Papez. Other areas of reduced gray matter volume included the right middle and superior temporal cortex, left precuneus and left medial frontal cortex [13,14].

Abnormalities in the fractional anisotropy of white matter tracts with diffusion-weighted magnetic resonance imaging (DWI-MRI) of the inferior longitudinal and inferior fronto-occipital fasciculi, right parietal white matter and the corpus callosum were detected in 48 Parkinson's disease (PD) patients with psychosis versus 42 PD patients without psychosis. The parietal white matter abnormalities may provide a putative explanation for haptic delusions in patients with DI [15].

As a side note, patients with schizophrenia show increased pre-synaptic dopamine activity in the cognitive striatum (caudate nucleus and dorsal putamen which have strong connections to frontal and parietal association cortex ) as opposed to the limbic striatum (the ventral pallidum and nucleus accumbens with strong connections to the hippocampus, amygdala and medial orbito-frontal cortex) [16].

When the brain is confronted with a stimulus, the stimulus is gauged by the motivational system (limbic striatum: nucleus accumbens, ventral pallidum) and assigned a value ("bits" of information) by the salience network (anterior cingular cortex, insular cortex). These systems are usually matched and gauged appropriately; in equipoise. A mismatch may attach an excessive value to a quanta of energy (the stimulus) and lead to excessive valuation of a stimulus and an inappropriate response. In a nutshell, this is the aberrant salience network hypothesis. [17,18].

Lastly, the glutamate N-methyl D-aspartate (NMDA) receptor is now well-established in the pathogenesis of psychosis. NMDA antagonists, such as phencyclidine (PCP) and ketamine produce psychotic behavior in humans. Their effects are dose-dependent and they are thought to reduce gamma-aminobutyric acid (GABA) inhibition, leading to excess acetylcholine and glutamate excitation [19].

Nowhere is this more dramatic as in the auto-immunity with anti-NMDA receptor encephalitis, where psychosis features prominently. This disease can be idiopathic, follow herpes simplex encephalitis or be a component of a paraneoplastic syndrome (ovarian teratoma). This encephalitis can also be associated with autonomic disorders, epileptic seizures and movement disorders [20].

## Conclusions

Undoubtedly psychosis is a fascinating condition that permeates clinical practice in the younger and older population. Its biology, in particular, its pharmacodynamics and neural circuitry is unfolding in an exciting way, as our therapeutic options continue to expand. Understanding the nuances of the circuitry and pharmacology of the agents used to treat psychosis will help us minimize adverse side-effects and improve patient quality-of-life and family satisfaction. We believe this article unravels and streamlines the complexities of psychosis by laying out the basics via a not unusual but under-reported clinical case scenario.
